# Patterns of dyslipidemia and its associated factors among prediabetic subjects. A cross-sectional study at a primary care clinic

**DOI:** 10.51866/oa.125

**Published:** 2023-12-29

**Authors:** Sania Siddiqui, Sabariah Noor Harun, Siti Maisharah Sheikh Ghadzi, Normala Abdul Wahid, Azima Binti Hassan, Hadzliana Zainal

**Affiliations:** 1 BPharm (Hons), MPharm (Clinical Pharmancy), PhD, School of Pharmaceutical Science, Universiti Sains Malaysia, Pulau Pinang, Malaysia. Email: hadz@usm.my; 2 MSc (Clinical Pharmacy), PharmD, School of Pharmaceutical Science, Universiti Sains Malaysia, Pulau Pinang, Malaysia.; 3 MSc (Clinical Pharmacy), PharmD, School of Pharmaceutical Science, Universiti Sains Malaysia, Pulau Pinang, Malaysia.; 4 BPharm (Hons), MPharm (Clinical Pharmacy), PhD, School of Pharmaceutical Science, Universiti Sains Malaysia, Pulau Pinang, Malaysia.; 5 MBBS, Pusat Sejahtera, Universiti Sains Malaysia, Pulau Pinang, Malaysia.; 6 BScN, Pusat Sejahtera, Universiti Sains Malaysia, Pulau Pinang, Malaysia.

**Keywords:** Prediabetic state, Dyslipidaemias, Patterns and factors, electronic health records

## Abstract

**Introduction:**

Diabetes is closely linked to cardiovascular diseases, with diabetic dyslipidaemia serving as an established marker of the acceleration of complications, contributing to an increased cardiovascular risk among patients. Timely detection and early characterization of lipid abnormalities can help clinicians in implementing effective preventive measures. This study aimed to determine the patterns and associated factors of dyslipidaemia among Malaysian subjects with borderline diabetes.

**Methods:**

A retrospective study was conducted among subjects with borderline diabetes aged ≥18 years who visited a primary healthcare centre at Universiti Sains Malaysia from January 2017 to December 2018. Sociodemographic, clinical and laboratory data were obtained from electronic medical records. Data were analysed using SPSS version 25.

**Results:**

A total of 250 participants with borderline diabetes were included in the analysis. Of them, 93.6% (n=234) had lipid abnormalities. Isolated dyslipidaemia characterised by a high low-density lipoprotein cholesterol (LDL-C) level (38.8%, n=97) was the most common pattern found, followed by combined dyslipidaemia of high LDL-C and triglyceride (TG) levels (22.8%, n=57). The male sex was found to be significantly associated with hypertriglyceridemia (adjusted odds ratio [AOR] = 1.86, 95% confidence interval [CI] =1.09–3.1)(P=0.02). Diastolic blood pressure ≥90mmHg was significantly associated with a low HDL-C level (A0R=2.09, 95% CI=1.0–4.1) (P=0.03).

**Conclusion:**

The majority of subjects with borderline diabetes have lipid abnormalities. Specifically, isolated dyslipidaemia characterised by a high LDL-C level is alarmingly prevalent. Further large-scale robust studies are needed to confirm the present findings.

## Introduction

Cardiovascular diseases (CVDs) are considered a leading cause of death in both developed and developing countries.^1^ Individuals with hyperglycaemia have a two- to four-fold increased risk of CVDs. Diabetic dyslipidaemia plays a critical role in the acceleration of macrovascular atherosclerosis and contributes to an increased risk of CVDs among patients with diabetes.^2^ Non-diabetic levels of hyperglycaemia, observed as impaired fasting glucose and impaired glucose tolerance, are also significantly associated with CVD morbidity and premature mortality.^3^ Epidemiological studies have shown that blood glucose levels in the prediabetic range are modestly correlated with many CVD risk factors including general and central obesity and increased blood pressure, triglyceride (TG), and lipoprotein levels.^4,5^ A prospective study with an 8-year follow-up of Mexican Americans without diabetes also documented higher levels of low-density lipoprotein cholesterol (LDL-C), TG, total cholesterol (TC), and blood pressure and lower levels of high-density lipoprotein cholesterol (HDL-C) among patients who subsequently developed diabetes than among individuals without diabetes.^2^ These findings confirm the presence of lipid abnormalities in the prediabetic state and indicate that patients with prediabetes have atherogenic patterns of CVD risk factors possibly owing to obesity, hyperglycaemia, and more importantly, insulin resistance. These atherogenic patterns are known to be present for many years and increase the risk of macrovascular complications as much as the duration of clinically defined diabetes itself.^2^ Thus, early recognition, screening, and management of dyslipidaemia among subjects with borderline diabetes are important to halt disease progression as well as prevent the development of atherogenic CVD events.

Although prediabetes has been associated with an increased risk of CVD events, the association observed is somewhat less than that with frank diabetes.^5,6^ Differences in the peak plasma level of glucose/insulin and/or in the lipid profile could also be a factor. A priorly published cohort study conducted among Chinese patients with hyperglycaemia also documented differences in the patterns of dyslipidaemia between subjects with borderline diabetes and with diabetes.^7^ The prevalence of low HDL-C levels was not substantially high, but the prevalence of more atherogenic LDL-C and TG was higher among subjects with borderline diabetes than among diabetic subjects.^7^ This pattern of dyslipidaemia is also quite commonly found among South Asian populations.^8^ However, no relevant studies have been conducted in Malaysia despite prediabetes affecting approximately 22.1% of adults aged ≥18 years in the country, with a high proportion (65.7%) of them having comorbid dyslipidaemia.^9^ The prevalence of dyslipidaemia among subjects with borderline diabetes is known to be high; however, available data regarding the patterns and associated factors of dyslipidaemia among Malaysian patients with borderline diabetes remain scarce. The limited knowledge about the status and patterns of dyslipidaemia may delay the implementation of effective treatment approaches for the prevention of lipid abnormalities and difficulties in the estimation of future CVD risks among patients with borderline diabetes.^10^ Accordingly, this retrospective study aimed to identify the patterns and associated factors of dyslipidaemia among adult Malaysian patients with borderline diabetes.

## Methods

### Study design andpopulation

A cross-sectional study via a retrospective review of medical records of patients with borderline diabetes was conducted at a primary healthcare centre at Universiti Sains Malaysia (USM). Ethical approval was obtained before the commencement of the study (reference no.: USM/JEPeM/18040197). The estimated sample size required for this study was calculated using the single-proportion formula,^11^ assuming a prevalence of dyslipidaemia of 65.7% among Malaysian patients with borderline diabetes^9^ after considering a 90% confidence interval (CI). Given the retrospective nature of the study and a targeted population meeting the inclusion criteria of a smaller size, 90% CI was used in this study to reach the targeted population with acceptable findings, accounting for an attrition rate of 10% and a precision of ±0.05. Based on the calculation, 244 patients were estimated to be required for the study.

A total of 250 patients who were aged ≥18 years, were diagnosed with borderline diabetes by a physician (plasma glucose levels above the cutoff values as recommended in the American Diabetes Association guidelines: fasting plasma glucose [FPG] level of 5.6–6.9 mmol/L and/or post-load plasma glucose level of 7.8–11.1 mmol/L and/or glycated haemoglobin level of 5.7–6.4%)^12^ and visited the study site from January 2017 to December 2018 were included in this study. Conversely, patients who were aged below 18 years; had type 1, type 2, gestational or steroid-induced diabetes; and had other causes of secondary dyslipidaemia such as hypothyroidism and other serious ailments such as myocardial infarction or stroke were excluded from this study.

### Data collection

Convenience sampling was used to recruit patients with borderline diabetes who satisfied the eligibility criteria. The registration records of 1892 patients receiving medical care from January 2017 to December 2018 were screened. The records of those diagnosed with borderline diabetes were further evaluated to retrieve relevant sociodemographic, clinical, and laboratory parameters. Data on patients’ sex, age, ethnicity, occupation, body mass index (BMI), FPG level, blood pressure and lipid profiles including TC, HDL-C, LDL-C and TG levels were extracted. Repeated inclusion of the same patients was avoided by using a filter based on their unique record of clinic number and full name. Dyslipidaemia was defined as TC, TG, LDL-C, and HDL-C levels above the cutoff values as recommended in the Malaysian guidelines for dyslipidaemia management; the optimal levels of TC, TG, and LDL-C are ≥5.2 mmol/L, ≥1.7 mmol/L and ≥2.6 mmol/L, respectively, while the optimal level of HDL-C is ≤1.0 mmol/L in men and ≤1.2 mmol/L in women.^13^ According to the patterns of dyslipidaemia, the patients were categorised into three types: 1) isolated dyslipidaemia, wherein any one of the lipid fractions is beyond the target level; 2) combined dyslipidaemia, wherein two lipid fractions are beyond the target level (i.e. high TG and LDL-C levels, high TG and low HDL-C levels and high LDL-C and low HDL-C levels); and 3) mixed dyslipidaemia, wherein more than two lipid fractions are beyond the target level (i.e. TG level of ≥1.7 mmol/L, LDL-C level of ≥2.6 mmol/L and HDL-C level of ≤1.0 mmol/L in men and ≤1.2 mmol/L in women). Conversely, the patients were considered to have hypertension when their recorded blood pressure was beyond the recommended range (i.e. systolic blood pressure of ≥140 mmHg and/or diastolic blood pressure [DBP] of ≥90 mmHg irrespective of their hypertensive treatment as suggested in the clinical practice guidelines for the management of hypertension).^14^ BMI was calculated as weight in kilograms divided by height in metres squared. Generalised obesity was defined using the BMI cutoff values for Asians mentioned in the clinical practice guidelines for the management of obesity in Malaysia. The patients were considered to be of normal weight with a BMI of <23 kg/m^2^, overweight with a BMI of ≥23 kg/m^2^, and obese with a BMI of ≥27.5 kg/m^2^.^15^

### Statistical analysis

Data were analysed using IBM SPSS statistics for windows, version 25. The Kolmogorov-Smirnov test was used to evaluate the normality of the data, confirming that the data were normally distributed. Continuous variables were reported as means and standard deviations and categorical variables as frequencies and percentages. Student’s t-test was utilised to compare continuous variables across the study groups. Continuous variables with more than two subcategories were compared using one-way between-group analysis of variance (ANOVA). Tukey's Honestly significant differences (HSD) post -hoc test was applied for OneWay ANOVA. Multiple imputations were used to handle variables with missing values above 10%. Missing values were found in the BMI of the participants, which were imputed via multiple imputation methods. Five imputations were used, and Rubin’s rules were implemented to combine the findings. Logistic regression analysis was conducted to predict the factors independently associated with dyslipidaemia among the subjects with borderline diabetes. Clinically relevant and statistically tested variables were included in the univariable regression analysis. Variables with a P-value of <0.25 were included in the multivariable analysis.

Correlation and multicollinearity between the independent variables were checked. The level of significance was set at P<0.05 for all tests.

## Results

### General characteristics of the participants

A total of 250 subjects with borderline diabetes were included in this study. Of them, 52.4% (n=131) were men, and 47.1% (n=119) were women. The mean age was 47.09±11.8 years, and the mean BMI was 27.0±5.54 kg/m^2^. The majority of the participants were middle-aged (<40 years), Malays (78%) and employed (80.4%). Dyslipidaemia was more common among men (n=122, 52.1%), Malays (n=184, 78.6%), and patients aged <40 years (n=90, 38.4%). Approximately 79.2% (n=198) of the participants were either overweight or obese, and 40.5% (n=95) of those who were overweight had dyslipidaemia. Comorbid hypertension was found among 47.6% (n=119) of the participants, among whom 48.7% (n=114) had abnormal lipid profiles (Table 1).

**Table 1:** General characteristics of the participants (N=250).

Variables	Categories	Overall n(%)	Dyslipidaemia n(%)		
			Yes (%)	No (%)	P-value
Sex	Male	131 (52.4%)	122 (52.1%)	9 (56.2%)	0.75
	Female	119 (47.6%)	112 (47.9%)	7 (43.8%)	
Ethnicity	Malay	195 (78.0%)	184 (78.6%)	11 (68.8%)	0.54
	Indian	37 (14.8%)	33(14.1%)	4 (25%)	
	Chinese	18 (7.2%)	17 (7.3%)	1 (6.2%)	
Age (year)	<40	92 (36.8%)	90 (38.5%)	2 (12.5%)	0.14
	41-50	51 (20.4%)	47 (20.1%)	4 (25%)	
	51-60	81 (32.4%)	74(31.6%)	7 (43-8%)	
	≥61	26(10.4%)	23 (9-8%)	3(18.7%)	
Occupation	Staff	201 (80.4%)	192 (82.0%)	9 (56.25%)	0.07
	Student	8 (3.2%)	7 (3.0%)	1 (6.25%)	
	Unemployed/pensioner	41 (16.4%)	35(15.0%)	6 (37.5%)	
BMI (kg/m^2^)	Normal	52 (20.8%)	48 (20.5%)	4 (25.0%)	0.78
	Overweight	100 (40.0%)	93 (39.7%)	7 (43.8%)	
	Obese	98 (39.2%)	93 (39.8%)	5(31.2%)	
Blood pressure status	Normotensive	131 (52.4%)	120 (51.3%)	11 (68.75%)	0.17
	Hypertensive	119 (47.6%)	114 (48.7%)	5(31.25%)	

The chi-square test was used to calculate the frequencies and percentages of the general characteristics of the participants according to the dyslipidaemia status. P<0.05 was considered statistically significant. BMI=body mass index

**Figure 1:**
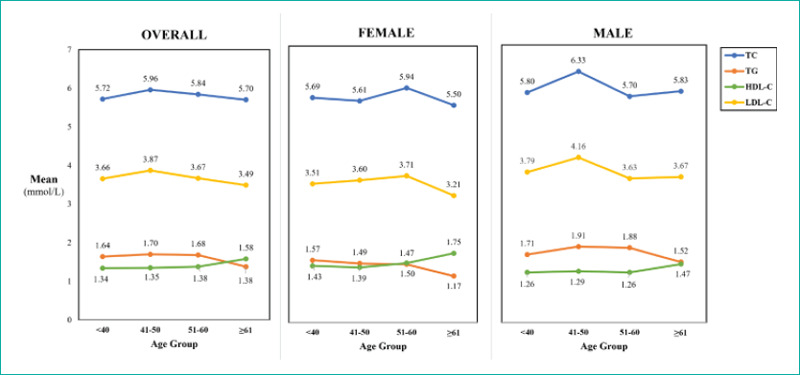
Sex- and age-specific mean lipid levels. Student’s t-test was used to compare the mean lipid levels according to sex. One-way ANOVA (Analysis of variance) was used to compare the mean serum lipid levels across the four age groups. Significant differences were observed in the serum HDL-C level across the age groups in one-way ANOVA (P<0.05) (Table S2). *Abbreviations;* TC: Total cholesterol; TG: Triglycerides; HDL-C; High-density lipoprotein cholesterol; LDL-C: Low-density lipoprotein cholesterol

The mean serum levels of the lipid parameters and their ratios with respect to age and sex were also calculated (Figure 1, Table S1 and Table S2). Student’s t-test was used to compare the mean lipid levels according to sex. The mean serum levels of all lipid parameters (except TCs) were significantly higher among the male patients than among the female patients (P<0.05). The mean serum HDL-C levels were significantly lower among the male patients (1.29±0.35 mmol/L) than among the female patients (1.47±0.40 mmol/L) (P<0.01) (Figure 1). One-way ANOVA followed by Tukey’s post hoc test was conducted to explore the effect of age on the mean serum levels. The analysis revealed that the mean serum HDL-C levels significantly differed across the four age groups (P<0.05). Tukey’s post hoc test demonstrated significant differences in the serum HDL-C level between the patients aged <40 and ≥61 years but no significant differences in the serum levels of the other lipid parameters. When the sample was stratified according to sex, no significant age group-specific variations in the mean serum lipid levels were observed between the male and female patients (P>0.05) (Figure 1 and Table S2).

### Patterns of dyslipidaemia

The patterns of dyslipidaemia among the male and female subjects with borderline diabetes are illustrated in Table 2. The most prominent lipid abnormality was isolated dyslipidaemia, affecting 38.8% (n=97) of the participants with a high LDL-C level. The least common lipid abnormality was a low HDL-C level, affecting 1.6% (n=4) of the participants. Combined dyslipidaemia was the second most common pattern of dyslipidaemia found, with high LDL-C and TG levels comprising the majority (22.8%, n=57) of this pattern, followed by high LDL-C and low HDL-C levels (14%, n=35) and high TG and low HDL-C levels (2.8%, n=7). The prevalence of combined dyslipidaemia of high LDL-C and TG levels was significantly higher among the male participants (29.8%, n=39) than among the female participants (15.1%, n=18) (P=0.006). In contrast, combined dyslipidaemia of high LDL-C and low HDL-C levels was significantly more prevalent among the female participants (20.2%, n=24) than among the male participants (8.4%, n=11) (P=0.007). No significant difference was observed in the other patterns between the male and female participants (P>0.05).

**Table 2:** Patterns of dyslipidaemia among subjects with borderline diabetes (N=250).

	Isolated dyslipidaemia			Combined dyslipidaemia			Mixed dyslipidaemia
	TG level of ≥1.7 mmol/L	HDL-C level of ≤1.0/1.2 mmol/L	LDL-C level of ≥2.6 mmol/L	LDL-C level of ≥2.6 mmol/L + TG level of ≥1.7 mmol/L	LDL-C level of ≥2.6 mmol/L + HDL-C level of ≤1.0/1.2 mmol/L	TG level of ≥1.7 mmol/L + HDL-C level of ≤1.0/1.2 mmol/L	LDL-C level of ≥2.6 mmol/L + TG level of ≥1.7 mmol/L + HDL-C level of ≤1.0/1.2 mmol/L
Men (n=131)	2 (1.5%)	1 (0.76%)	51 (38.9%)	39 (29.8%)	11 (8.4%)	3 (2.3%)	15 (11.5%)
Women (n=119)	5 (4.2%)	3 (2.5%)	46 (38.6%)	18 (15.1%)	24 (20.2%)	4 (3.4%)	12 (10.1%)
Total (N=250)	7 (2.8%)	4 (1.6%)	97 (38.8%)	57 (22.8%)	35 (14.0%)	7 (2.8%)	27 (10.8%)
P-value	0.18	0.27	0.72	**0.006**	**0.007**	0.89	0.7

Data are expressed as frequencies and percentages. TG=triglyceride; HDL-C=high-density lipoprotein cholesterol; LDL-C=low-density lipoprotein cholesterol

### Factors associated with dyslipidaemia

In the multivariable logistic regression analysis (Table 3), the male sex was found to be significantly (P=0.02) associated with hypertriglyceridaemia (adjusted odds ratio [AOR] = 1.86, 95% CI=1.09–3.1). The risk of having low HDL-C levels was significantly higher among the male participants than among the female participants (AOR=0.57, 95% CI=0.2–0.9) (P<0.05). The DBP was significantly associated with a low HDL-C level (A0R=2.09, 95% CI=1.0–4.1) (P=0.03). No significant association of the other variables with high TG, TC, and LDL-C levels was observed among the participants (P>0.05).

**Table 3:** Univariable and multivariable logistic regression analyses of the risk factors associated with various categories of dyslipidaemia among the subjects with borderline diabetes.

	High TC level				High TG level			
	Univariable		Multivariable		Univariable		Multivariable	
	COR (95% CI)	P-value	AOR (95% CI)	P-value	COR (95% CI)	P-value	AOR (95% CI)	P-value
Male sex	1.28 (0.7-2.2)	0.37	--	--	1.80 (1.1-3.1)	0.01	1.86 (1.09-3.1)	0.02
Age of >40 years	1.14 (0.6-2.0)	0.65	--	--	1.15 (0.6-1.9)	0.59	--	--
BMI of >23 kg/m^2^	0.58 (0.2-1.2)	0.16	0.58 (0.2-1.2)	0.16	0.88 (0.4–1.6)	0.69	--		--
Malay ethnicity	1.29 (0.6-2.5)	0.43	--	--	1.49 (0.7-2.8)	0.22	1.44 (0.7-2.8)	0.28
Unemployment	1.2 (0.6-2.2)	0.55	--	--	0.68 (0.3-1.2)	0.19	0.81 (0.4-1.4)	0.49
SBP of >140 mmHg	1.59 (0.8-2.8)	0.12	1.6 (0.8-2.9)	0.11	0.98 (0.5-1.6)	0.94	--	--
DBP of >90 mmHg	1.34 (0.6-2.6)	0.38	--	--	1.36 (0.7-2.4)	0.3	--	--
FPG level	0.98 (0.9-1.0)	0.21	0.98 (0.9-1.0)	0.19	1.0 (0.9-1.02)	0.96	--	--
	Low HDL-C level				High LDL-C level High TG level			
	Univariable		Multivariable		Univariable		Multivariable	
	COR (95% CI)	P-value	AOR (95% CI)	P-value	COR (95% CI)	P-value	AOR (95% CI)	P-value
Male sex	0.61 (0.3-1.0)	0.08	0.57 (0.2-0.9)	0.02	1.1 (0.5-2.3)	0.77	--	--
Age of ≥40 years	0.57 (0.3-1.01)	0.05	0.71 (0.3-1.3)	0.27	0.56 (0.2-1.3)	0.20	0.56 (0.2-1.3)	0.20
BMI of ≥23 kg/m^2^	1.15 (0.5-2.2)	0.68	--	--	0.73 (0.2-2.0)	0.55	--	--
Malay ethnicity	1.27 (0.6-2.5)	0.49	--	--	1.33 (0.5-3.1)	0.51	--	--
Unemployment	0.57 (0.3-1.07)	0.08	0.58 (0.2-1.1)	0.11	0.64 (0.2-1.4)	0.27	--	--
SBP of ≥140 mmHg	0.68 (0.3-1.2)	0.19	0.57 (0.3-1.0)	0.08	1.13 (0.5-2.4)	0.76	--	--
DBP of ≥90 mmHg	1.62 (0.8-2.99)	0.11	2.09 (1.0–4.1)	0.03	1.09 (0.4–2.6)	0.84	--	--
FPG level	1.0 (0.9-1.04)	0.20	1.02 (0.9–1.0)	0.09	0.9 (0.9–1.02)	0.57	--	--

AOR=adjusted odds ratio; BMI=body mass index; CI=confidence interval; COR=crude odds ratio; DBP=diastolic blood pressure; FPG=fasting plasma glucose; SBP=systolic blood pressure; TC=total cholesterol; TG=triglyceride; HDL-C=high-density lipoprotein cholesterol; LDL-C=low-density lipoprotein cholesterol

## Discussion

The majority of the participants had LDL-C levels higher than the recommended range in the Malaysian guidelines for dyslipidaemia management.^13^ Herein, the most and least common lipid abnormalities were a high LDL-C level and a low HDL-C level, respectively. Similar findings were reported in previous studies conducted among Southeast Asian^10^ and Middle Eastern^16^ patients with hyperglycaemia, wherein the most frequent form of dyslipidaemia was a high LDL-C level with a frequency of 48.3% and 49%, respectively. High LDL-C levels (≥2.6 mmol/L) are known to pose a substantial risk for atherogenesis and the development of near-future CVD and coronary heart disease events.^17^ Similarly, low HDL-C levels (<1.0 mmol/L) are known to play a pivotal role in the atherogenic process. The coexistence of these two lipid abnormalities affected nearly 14% of the participants in this study, with the women (20.2%) being more affected than the men (8.4%). The prevalence of combined dyslipidaemia of high LDL-C and TG levels was significantly higher among the men than among the women (29.8% vs 15.1%). These findings are consistent with those of the study conducted among Nepalese patients with type 2 diabetes with a known prevalence of combined dyslipidaemia of high LDL-C and TG levels, which was significantly higher among men than women.^18^ In the present study, mixed and combined dyslipidaemia were observed in only fractions of the participants. Similarly, studies conducted among African^19^ and Southeast Asian subjects with borderline diabetes^20^ showed that mixed dyslipidaemia was present in only 16% and 17.6%, respectively. Co-existing lipid abnormalities along with insulin resistance and hyperglycaemia for a longer duration may increase the risk of CVDs, suggesting the need to rectify such abnormalities at the initial stage.

The mean serum levels of all lipid parameters (except HDL-C) were significantly higher among the male subjects with borderline diabetes than among their female counterparts in this study. This finding agrees with that in studies conducted among Chinese,^7^ Iranian^21^ and Indian^22^ subjects with borderline diabetes, wherein the mean serum levels of LDL-C and TG were higher, and the mean serum level of HDL-C was lower among male patients than among female patients. Differences in the sex hormones and body fat distribution between men and women could explain such discrepancies in the lipid profiles. The high prevalence among men may also be attributed to the lack of a cardio-protective effect of the female sex hormone, high visceral body fat distribution accompanied by reduced lipid metabolism and lipoprotein kinetics among men.^23^

As age is a non-modifiable risk factor of CVDs,^24^ its effect on the serum lipid profile was also evaluated in this study across the four age groups using one-way ANOVA. A significant difference was observed only in the serum HDL-C level between the patients aged <40 and ≥61 years, irrespective of sex. This finding is comparable with other reports.^18,20^ Further, the lipid levels among the participants were noted to increase with age, peaked at the age of 51–60 years and declined beyond the age of 60 years. Similarly, a previous review documented that the serum lipid levels (including TC and LDL-C) among older adults from 10 different countries^25^ were notably increased from the age of puberty to the age of 55 years, followed by a decline beyond the age of 60 years. The effect of such decline in the mean lipid levels with advancing age (beyond 60 years) could be attributed to a reduction in the cholesterol synthesis owing to the decline in the liver function with increasing age.^25^ Conversely, the HDL-C level increases with increasing age. Body weight and eating habits mainly including dietary fat intake may have a significant effect on HDL-C levels. Body weight has been reported to decline with age among Malaysian patients.^26^ Shift of dietary fat intake from saturated to non-saturated fatty lipids relative to increasing age could also explain the higher HDL-C levels among older adults than among young adults.^26^

The risk factors associated with dyslipidaemia were also evaluated in this study using multivariable analysis. A high TG level was found to be significantly associated with the male sex. This finding is consistent with other reports that male patients had significantly high LDL-C and non-HDL-C levels.^18^ The high TG and LDL-C levels among men could be explained in part by the differences in the sex hormones and the central fat distribution between men and women.^27^ Oestrogen generally reduces the circulating TG and LDL-C levels but increases the HDL-C level, resulting in the inherited cardio-protective effect in women.^28^ Differences in the lipid metabolism and kinetics of lipoprotein also account for the sexual dimorphism in the plasma lipid levels between sexes.^28^ Women generally have a strong anti-inflammatory immune profile that acts as a compensatory mechanism to limit increases in the blood pressure, ultimately helping control dyslipidaemia.^28^ Women are also metabolically inclined to store fat in subcutaneous tissues rather than in the abdominal region.^24^ In contrast, men tend to store adipose fat preferentially in visceral tissues and the abdominal region. A high proportion of fat as visceral adipose tissue is known as a significant predictor of dyslipidaemia.^23^ This could explain why the men were more susceptible to dyslipidaemia than the women in our study. The women tended to show a good lipid profile apart from the low HDL-C level, which was significantly associated with the female sex. This might be linked to several other factors such as the onset of menopause, which mimics low HDL-C levels among women, or the intake of high-fat diet or low level of physical activity.^29^ However, the actual association remains uncertain, as these confounders were not evaluated in this study. A low HDL-C level was strongly associated with an increased DBP in this study. Similarly, a large population-based study reported that the HDL-C and total serum cholesterol levels were independently and positively associated with the DBP.^30^ This effect may be attributed to the insulin resistance already established in the prediabetic state, significantly contributing to increases in visceral adiposity, hypertension, glucose intolerance, and ultimately, dyslipidaemia.^24^

### Limitations

The present study has some limitations. First, the study was conducted at a single healthcare centre in Malaysia, limiting the generalisability of the findings to the whole Malaysian population with borderline diabetes. Second, the study did not analyse the types and effects of lipid-lowering treatment among patients with dyslipidaemia. Data on antihypertensive drugs were not included, as this retrospective study depended mainly on data obtained from patient records. The findings might have been confounded by other factors, such as nutrition, physical activity, and concomitant morbidities. This aspect should be taken into account by future studies.

## Conclusion

A high LDL-C level was the most common pattern of dyslipidaemia, followed by high LDL-C and TG levels among the subjects with borderline diabetes. A high TG level was associated with the male sex, while a low HDL-C level was strongly associated with the female sex, suggesting a high risk of future CVDs among these populations. These findings highlight the extensive need for early screening of the lipid profiles of subjects with borderline diabetes. Effective interventions and targeted treatment approaches should also be implemented by healthcare professionals to prevent poor cardio-metabolic profiles and achieve optimum care.

## Data sharing statement

The datasets used and/or analysed during the current study are available from the corresponding author upon reasonable request.

**How does this paper make a difference in general practice?**

- Diabetic dyslipidaemia is a major public health problem in developing countries and an independent predictor of cardiovascular diseases.
- The findings provide useful insights into dyslipidaemia patterns commonly found among subjects with borderline diabetes.
- High low-density lipoprotein cholesterol levels alone and in combination with high triglyceride levels are the most common patterns found.
- High triglyceride levels and low high-density lipoprotein cholesterol levels are associated with the male and female sexes, respectively, suggesting a high risk of future cardiovascular diseases among them.
- This study provides baseline findings that may help clinicians in deciding and implementing targeted treatment approaches to prevent poor metabolic profiles.
